# Directional association test reveals high-quality putative cancer driver biomarkers including noncoding RNAs

**DOI:** 10.1186/s12920-019-0565-9

**Published:** 2019-12-30

**Authors:** Hua Zhong, Mingzhou Song

**Affiliations:** 1 Department of Computer Science, New Mexico State University, University Ave, Las Cruces, 88003 NM USA; 2 Molecular Biology Graduate Program, New Mexico State University, University Ave, Las Cruces, 88003 NM USA

**Keywords:** FunChisq, Non-monotonic directional association, Human cancer, Cancer driver gene, Noncoding RNA, Leukemia, Biomarker

## Abstract

**Background:**

Most statistical methods used to identify cancer driver genes are either biased due to choice of assumed parametric models or insensitive to directional relationships important for causal inference. To overcome modeling biases and directional insensitivity, a recent statistical functional chi-squared test (FunChisq) detects directional association via model-free functional dependency. FunChisq examines patterns pointing from independent to dependent variables arising from linear, non-linear, or many-to-one functional relationships. Meanwhile, the Functional Annotation of Mammalian Genome 5 (FANTOM5) project surveyed gene expression at over 200,000 transcription start sites (TSSs) in nearly all human tissue types, primary cell types, and cancer cell lines. The data cover TSSs originated from both coding and noncoding genes. For the vast uncharacterized human TSSs that may exhibit complex patterns in cancer versus normal tissues, the model-free property of FunChisq provides us an unprecedented opportunity to assess the evidence for a gene’s directional effect on human cancer.

**Results:**

We first evaluated FunChisq and six other methods using 719 curated cancer genes on the FANTOM5 data. FunChisq performed best in detecting known cancer driver genes from non-cancer genes. We also show the capacity of FunChisq to reveal non-monotonic patterns of functional association, to which typical differential analysis methods such as *t*-test are insensitive. Further applying FunChisq to screen unannotated TSSs in FANTOM5, we predicted 1108 putative cancer driver noncoding RNAs, stronger than 90% of curated cancer driver genes. Next, we compared leukemia samples against other samples in FANTOM5 and FunChisq predicted 332/79 potential biomarkers for lymphoid/myeloid leukemia, stronger than the TSSs of all 87/100 known driver genes in lymphoid/myeloid leukemia.

**Conclusions:**

This study demonstrated the advantage of FunChisq in revealing directional association, especially in detecting non-monotonic patterns. Here, we also provide the most comprehensive catalog of high-quality biomarkers that may play a causative role in human cancers, including putative cancer driver noncoding RNAs and lymphoid/myeloid leukemia specific biomarkers.

**Electronic supplementary material:**

The online version of this article (10.1186/s12920-019-0565-9) contains supplementary material, which is available to authorized users.

## Background

Greatly outnumbering coding genes, noncoding RNA (ncRNA) genes remain elusive in our understanding of their function. Among various ncRNAs, microRNA, long noncoding RNA, and enhancer RNA are the most heavily studied and some are deregulated in cancer [1–3]. Due to technical challenges caused by their typically low abundance, ncRNA profiles of cancer are yet widely available. For example, even in The Cancer Genome Atlas (TCGA) project [4], the expression of non-polyadenylated ncRNAs in tumor samples is not provided. Encouragingly, the Functional Annotation of Mammalian Genome 5 (FANTOM5) project [5] measured promoter-level transcriptome data at 209,911 transcription start sites (TSSs) in 752 human samples covering all major human tissue types, primary cell types, and notably many cancer cell lines represented by 225 samples. Such a sampling diversity captured a wealth of system dynamics. Additionally, technical variations introduced in data acquisition are minimal because all samples in the project were sequenced at the same facility housed in RIKEN, Japan. More than half (107,139) of the TSSs are unannotated, pointing to most likely novel ncRNAs. Therefore, the FANTOM5 data set opens up an enormous opportunity to study the role for ncRNAs in cancer.

Most statistical methods used to identify cancer marker genes [6, 7] are either biased due to parametric model choices, insensitive to directional causal relationships, or unable to reveal non-monotonic patterns. Table 1 summarizes advantages and disadvantages of several widely used biomarker detection methods. A symmetric association test reveals no directionality of a pattern, and thus cannot infer causality. Differential gene expression analysis methods are often unable to detect non-monotonic patterns from gene to phenotype, commonly seen in biological systems. Logistic regression can fit a nonlinear function but requires a correct parametric model. To overcome these issues, the functional chi-squared test (FunChisq) [8–10] is a recently developed statistical test for directional association via model-free functional dependency. The FunChisq test statistic is computed from a contingency table, where the row variable represents independent variable *X* and the column variable for dependent variable *Y*. When both *X* and *Y* are numeric or ordinal, we can define the monotonicity of a pattern. *X* to *Y* is monotonically increasing/decreasing if *Y* never decreases/increases as *X* increases. *X* to *Y* is non-monotonic if *Y* can both increase at one point and decrease at another as *X* increases. The FunChisq test statistic is maximized by either one-to-one or many-to-one non-constant functions from *X* to *Y* given marginal sums of dependent variable *Y*. Thus, FunChisq is sensitive to both monotonic and non-monotonic functional patterns. The original FunChisq test established an asymptotic chi-squared null distribution for the test statistic [8]. An exact functional test using the same test statistic has been developed to compute its statistical significance based on an exact, instead of asymptotic, null distribution [9]. We also introduce function index *ξ*_*f*_, derived from the FunChisq statistic, to measure the effect size of functional dependency. The relationship of the index to the *p*-value of the FunChisq test statistic is analogous to that of fold-change to *p*-value in differential gene expression analysis. The pair of fold change and *p*-value is often visualized together in a volcano plot. Similarly, examining both the function index and the FunChisq *p*-value disfavors patterns either weak in functional dependency or statistically insignificant, leading to increased confidence in causal inference.

**Table 1:** Comparison of widely used biomarker detection methods

Methods	Advantages	Disadvantages
Pearson’s chi-squared test	Model free	No directionality
*t*-test	No discretization	No non-monotonicity
Wilcoxon test	Nonparametric	No non-monotonicity
Logistic regression	Nonlinear No discretization	Requires a parametric model
DESeq2; edgeR	Generalized linear model	Requires a parametric model

The Heritage Provider Network (HPN)-Dialogue for Reverse Engineering Assessments and Methods (DREAM) network inference challenges aimed to decipher causal gene networks connecting signaling proteins in human breast cancer [11]. It evaluated network inference approaches employed or designed by about 80 participating teams for their effectiveness on revealing signaling networks. On the *in silico* data from a non-linear dynamical system model, FunChisq performed the best among all submissions. On the experimental phosphoprotein data measured from cancer cell lines in response to stimuli, prior biological knowledge about molecular interactions was allowed to be integrated. Notably, FunChisq, without incorporating any prior information, was ranked the 7th after six methods all using prior knowledge. In the post-challenge evaluation, combining prior knowledge with FunChisq led to substantial better performance over the best performer on the experimental data [11]. The outstanding performance of FunChisq supports its practicality in causal inference. Its advantage in distinguishing interaction directionality and sensitivity to non-monotonic patterns motivated us to study genes involved in cancer using FANTOM5 data.

On FANTOM5 data, we first evaluated FunChisq and six other methods using 719 curated cancer genes. FunChisq performed best in detecting known cancer driver genes from non-cancer genes. We also show the capacity of FunChisq to reveal non-monotonic patterns, to which typical differential analysis method such as *t*-test are insensitive. We further applied FunChisq on unannotated human TSSs in FANTOM5, and predicted 1108 ncRNAs as putative cancer drivers. They have directional association to cancer stronger than 90% of the curated cancer driver genes. Next, we compared leukemia samples against other samples in FANTOM5 and FunChisq predicted potential biomarkers for lymphoid leukemia and for myeloid leukemia, stronger than all known driver genes of the two leukemia types.

This study demonstrates that FunChisq indeed detected many non-monotonic TSS-cancer association patterns, to which previous methods may be blind. As the TSS-cancer associations are predicted by directional functional dependency without assuming a parametric model, we have provided the most comprehensive and unbiased catalog of high-quality noncoding and coding RNA TSSs that may be causative factors to human cancers.

## Results

### FunChisq is powerful in detecting known human cancer genes

We evaluated the performance of FunChisq and six other tests in distinguishing 719 curated cancer genes on FANTOM5 human data. The six other tests include Pearson’s chi-squared test [12], Wilcoxon test [13], *t*-test [14], logistic regression [15], DESeq2 [16], and edgeR [17]. The curated cancer genes were obtained from Cancer Gene Census [18] in COSMIC Release v83. The ground truth in the evaluation was generated with true cancer driver genes and non-cancer-associated genes. For each cancer driver gene, we extracted its representative TSS, which was the most transcribed among all TSSs of the same gene. However, non-cancer-associated genes are not typically reported in the literature. Thus, excluding curated cancer genes, we randomly picked the same number of TSSs—most likely non-cancer TSSs. Then we evaluated all seven methods for their performance in revealing true cancer driver gene TSSs. DESep2 and edgeR were tested on raw read count data, while the other methods on discrete data transformed from expression data in the unit of tags per million (TPM). Specifically, we used the R package *Ckmeans.1d.dp* [19, 20] to discretize the log-transformed TPM abundance from all samples for each TSS, before which numbers of discretization levels for each gene were automatically determined by R package *mclust* [21] by fitting a finite Gaussian mixture model.

**Fig. 1:**
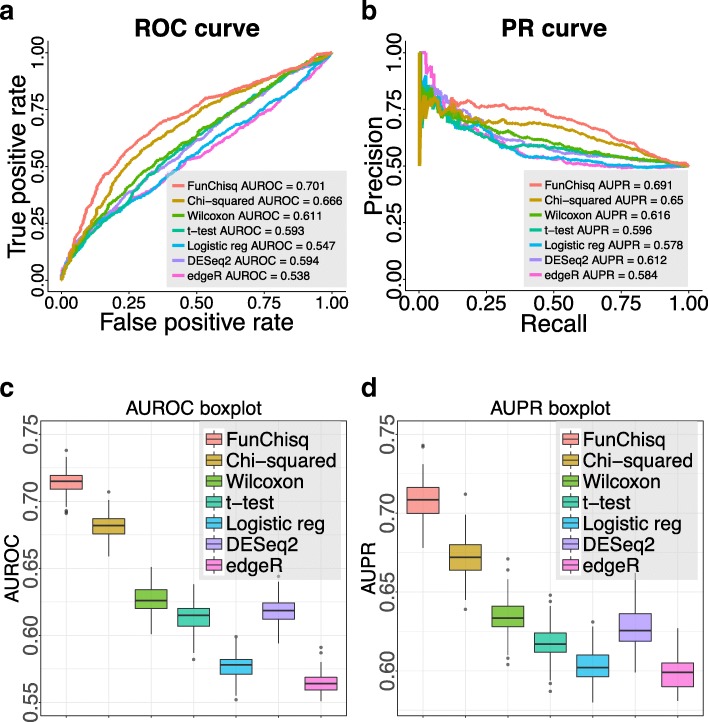
FunChisq outperformed six widely-used methods in detecting known cancer genes from FANTOM5 data. FunChisq test, Pearson’s chi-squared test, Wilcoxon test, *t*-test and logistic regression used transformed expression data. DESeq2 and edgeR used raw read count data. **a** ROC curves of each method. **b** PR curves of each method. **c** AUROC distributions after repeating the randomized evaluation 100 times. **d** AUPR distributions after repeating the randomized evaluation 100 times

The performance of the seven methods on detecting cancer TSSs from FANTOM5 data is summarized in Fig. 1. The receiver operating characteristic (ROC) curves in Fig. 1a and precision-recall (PR) curves in Fig. 1b indicate that FunChisq outperformed the other six methods. We repeated the same evaluation on 100 different sets of randomly selected non-cancer TSSs. Figure 1c,d show that the areas under the ROC and PR curves of FunChisq are markedly better than all other six methods, demonstrating the advantage of FunChisq. The fact that directional FunChisq scored better than directionless Pearson’s chi-squared test suggests the importance of direction in detecting cancer genes. FunChisq also performed much better than the other five methods (Wilcoxon test, *t*-test, DESeq2, edgeR, and logistic regression) not designed for detecting non-monotonic patterns, suggesting the importance of detecting such patterns when analyzing cancer driver gene expression, as demonstrated in the next subsection.

### FunChisq is sensitive to non-monotonic patterns

**Fig. 2:**
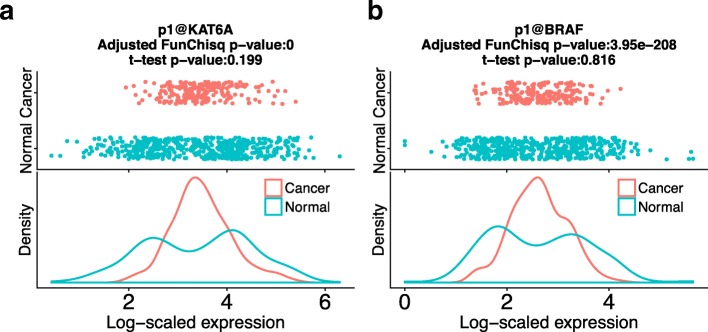
Non-monotonic directional interaction patterns from two known cancer genes to the cancer status of human samples. The horizontal axes are log-scaled abundance of the most expressed TSS of each gene from FANTOM5. The vertical axes of the two top plots represent tissue types. ‘Cancer’ indicates a sample is from a cancer cell-line, ‘Normal’ for a sample from a non-cancer tissue. The vertical axes of the two bottom plots are the probability density of gene expression level. FunChisq reported high statistical significance of both genes’ directional association with cancer suggested by the low *p*-values, while *t*-test returned insignificant results indicated by large *p*-values. **a***p1@KAT6A*, the most transcribed TSS of known cancer gene *KAT6A*, is either up- or down-regulated in 527 non-cancer samples of various tissues, but has an intermediate level of expression in 225 samples of various cancers. **b***p1@BRAF*, the most transcribed TSS of known cancer gene *BRAF*, has a similar non-monotonic expression profile directionally associated with cancer status

On the whole-body FANTOM5 human transcriptome data, we showcase non-monotonic interaction patterns between TSS abundance of two known cancer genes, *KAT6A* (also known as *MYST3* and *MOZ*) [22] and *BRAF* [23], and their cancer status of human samples in Fig. 2. The non-monotonicity was detected only by FunChisq, while approaches based on comparison of means, such as *t*-test, would fail, because the means of non-monotonic patterns between cancer and non-cancer samples may not differ significantly. KAT6A has been implicated to either promote or inhibit senescence [24], important for tumor formation and growth [25]. KAT6A is associated with oncogenesis [22] in both leukemia [26–29] and breast cancer [30], because of dysregulation of its histone acetyltransferase activity or its aberrant expression. KAT6A was also hypothesized to suppress tumor when severe DNA damage happened [24, 31]. Thus, KAT6A may both promote and suppress cancer, playing competing roles depending on the cellular context. BRAF has long been established as a proto-oncogene [32]. However, BRAF paradoxically inhibits stem cell renewal [33]; also in BRAF-driven mouse model of colon cancer, tumor formation is suppressed [33]. Therefore, BRAF may either promote or inhibit cancer depending on the context. Both examples illustrate the capacity of FunChisq in recognizing non-monotonic patterns, which *t*-test and other statistical analysis methods based on the comparison of group means may not manage to differentiate.

### FunChisq is empirically efficient in runtime

We measured the total runtime of the seven methods evaluating the relationship of all TSSs to cancer, as summarized in Table 2. The input to each method is the FANTOM5 data covering 209,911 TSSs across 752 samples, including 527 cancer cell lines and 225 normal primary/tissue cells. The program ran on a single thread of a server with 12 ×2.40GHz Intel(R) Xeon(R) CPU E5645 and 192GB RAM under openSUSE Leap 15.0 OS. FunChisq, Pearson’s chi-squared test, Wilcoxon test and *t*-test took the least time of less than 10 minutes. Logistic regression and edgeR took much longer time fitting default models. DESeq2 costed most time due to raw read count normalization, dispersion estimation, and generalized linear model fitting. In summary, the empirical runtime comparison suggests that FunChisq is practically efficient.

**Table 2:** Empirical runtime of seven methods in evaluating association of 209,911 transcription start sites with cancer

Methods	Runtime
*t*-test	2m 26s
Pearson’s chi-squared test	8m 32s
FunChisq	8m 40s
Wilcoxon test	8m 41s
edgeR	43m 44s
Logistic regression	44m 01s
DESeq2	54h 08m

The methods are sorted in the increasing order of runtime

### FunChisq reveals putative cancer driver noncoding rNAs

**Fig. 3:**
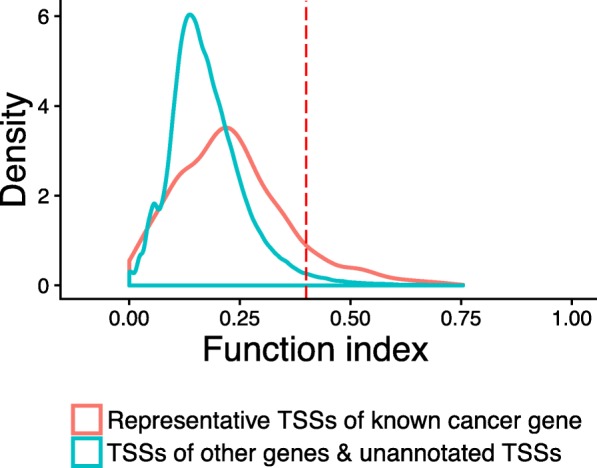
Distributions of function index measuring the directional association from TSSs to cancer status. The red curve is the distribution of the index from representative TSSs of known cancer genes to cancer status. The blue curve is the distribution of representative TSSs of non-cancer genes to cancer status. Cancer gene TSSs apparently have more larger index values than non-cancer gene TSSs, implying that the former group is more powerful than the latter group at predicting cancer status. About 90% of known cancer gene representative TSSs have an index value of less than 0.40, as indicated by the vertical red dashed line

The latest FANTOM5 annotation has identified most coding genes in the human genome. Thus, we hypothesize that the majority of the 107,139 unannotated TSSs may belong to potential novel ncRNAs. To identify the directional effect from TSS to cancer, we applied FunChisq on the expression of each TSS in cancer versus non-cancer samples to report function indices and *p*-values. Figure 3 shows the distribution of function index of representative TSSs from the 719 known cancer genes, versus that of all other TSSs. The two distributions demonstrate that known cancer TSSs have a greater average function index than other TSSs, indicating that the cancer status has stronger dependency on known cancer TSSs than other TSSs.

**Fig. 4:**
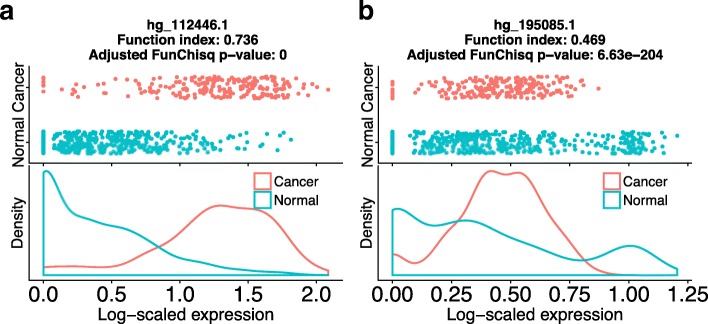
Two unannotated transcription start sites predicted as putative cancer driver ncRNAs. The horizontal axes are log-scaled TSS expression from FANTOM5. The vertical axes of the two top plots represent tissue types. ‘Cancer’ indicates a sample is from a cancer cell-line, ‘Normal’ for a sample from a non-cancer tissue. The vertical axes of the two bottom plots are the probability density of gene expression level. **a** Putative cancer ncRNA *hg _112446.1* has a monotonic pattern with cancer status. **b** Putative cancer ncRNA *hg _195085.1* exhibits a non-monotonic pattern with cancer status

Rather than picking a fixed function index cutoff, we selected the threshold at 90 percentile of known cancer TSS function index values (Fig. 3). The criterion is stringent to select the most relevant candidates. At the 90 percentile function index cutoff of 0.40 and an adjusted *p*-value threshold of 0.05, we selected 1108 unannotated TSSs with a directional effect on cancer status. Thus they are stronger than 90% of representative TSSs of all known cancer driver genes, constituting putative cancer driver ncRNAs. Figure 4 shows two such predicted ncRNAs, one with a monotonic interaction pattern with cancer status and the other a non-monotonic pattern. All 1108 predicted noncoding cancer TSSs are listed in Additional file 1. We expect cancer biologists to find these ncRNA biomarkers interesting and to apply either RNA silencing or gene editing to study their functions in cancer.

### Putative cancer-type specific biomarkers for lymphoid and myeloid leukemias

Both lymphoid and myeloid leukemia samples have the largest sample size among all cancer types sequenced by the FANTOM5 project. We contrast samples of a cancer type and all remaining samples which also include other cancer types, such that the markers identified are only specific to the cancer type of interest. This strategy is only possible with FANTOM5 data in that they cover all major tissue, cell, and cancer types in human.

We first searched for potential biomarkers of lymphoid leukemia by testing the directional effect of each TSS on lymphoid leukemia status. Among all 752 samples from FANTOM5, there are 23 lymphoid leukemia and 48 related normal lymphoid samples. We divided the samples into two groups: the first group contains 23 lymphoid leukemia samples and the second group has all other 729 samples (including the 48 normal lymphoid samples and all cancer types other than lymphoid leukemia). We then performed the FunChisq test on each TSS to hunt for ones on which lymphoid leukemia status functionally depend. By requiring a *p*-value under 0.05 and a function index greater than all 87 known lymphoid leukemia driver gene TSSs, we identified 332 putative lymphoid leukemia biomarkers.

Next we performed the same procedure to search for biomarkers for myeloid leukemia by contrasting the 28 myeloid leukemia samples with the remaining 724 samples (including 26 normal myeloid samples and all cancer types other than myeloid leukemia). We detected 79 statistically significant putative myeloid leukemia biomarkers, with a *p*-value no more than 0.05 and function index greater than the TSSs of all 100 known myeloid leukemia driver genes.

**Fig. 5:**
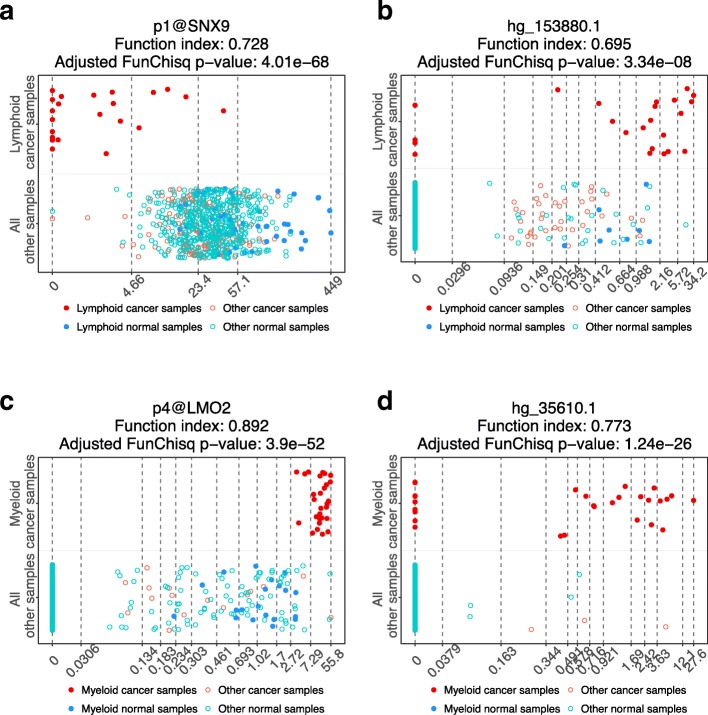
Gene expression patterns of four potential leukemia biomarkers are nearly exclusively cancer-type specific. The horizontal axes are TSS levels of gene expression from FANTOM5. The vertical axes are sample types. **a** Putative lymphoid leukemia biomarker *SNX9*. **b** Putative lymphoid leukemia biomarker *hg _153880.1*. **c** Putative myeloid leukemia biomarker *LMO2*. **d** Putative myeloid leukemia biomarker *hg _35610.1*

Figure 5 illustrates the expression patterns of four biomarker candidates that are distinct between the specific leukemia and other samples. Only in lymphoid leukemia, *p1@SNX9* is under-expressed but not in any other samples (Fig. 5a); *hg_153880.1* is mostly highly expressed only in lymphoid leukemia (Fig. 5b). *p4@LMO2* is exclusively highly expressed in myeloid leukemia (Fig. 5c); *hg_35610.1* also exhibited the highest expression in myeloid leukemia (Fig. 5d).

**Fig. 6:**
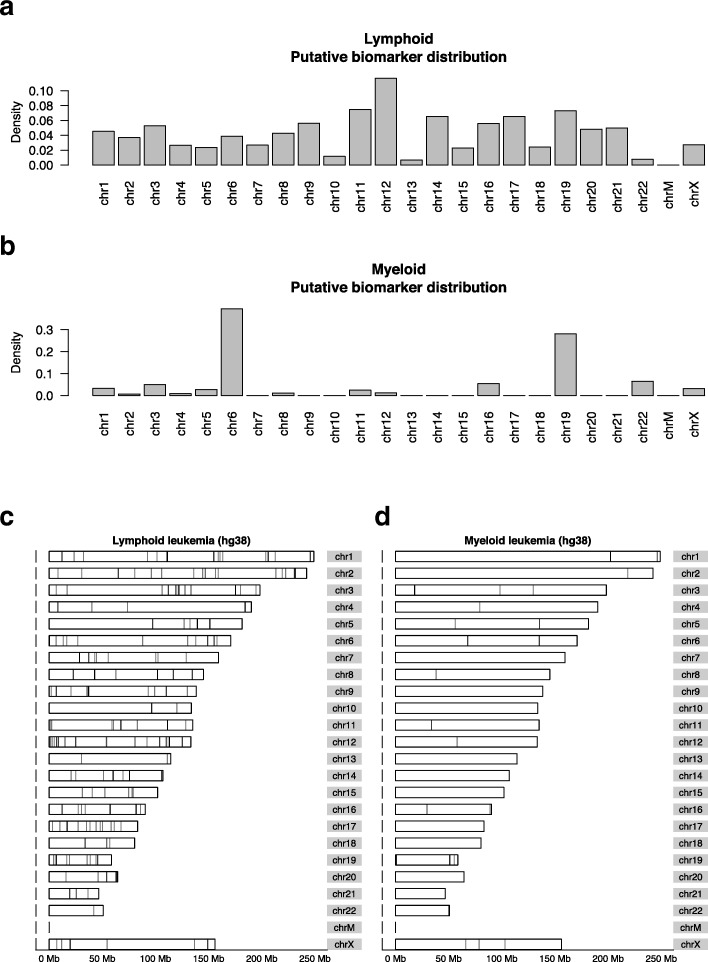
Chromosomal locations of putative leukemia biomarkers. Chromosomal counts of putative biomarkers for (**a**) lymphoid and (**b**) myeloid leukemia. Genomic maps of putative biomarkers for (**c**) lymphoid and (**d**) myeloid leukemia

Distributions of detected biomarkers along each chromosome for lymphoid and myeloid leukemias are shown in Fig. 6. In lymphoid leukemia samples, chromosomes 12 contain the highest number of biomarkers, while in myeloid leukemia samples, chromosome 6 and 19 has much more biomarkers than others. In chronic lymphocytic leukemia (CLL), trisomy 12 has been reported to be the third most frequent chromosomal aberration and is often present as a unique cytogenetic alteration [34]. In acute myeloid leukemia (AML), trisomy chromosome 6 has been reported as a sole cytogenetic abnormality in AML-M5 [35], and chromosome 19 abnormalities are commonly seen in AML-M7 [36]. Our findings of the biomarker genomic locations are consistent with these known chromosomal abnormalities in subtypes of leukemia, which supports potential cancer-related functions of the putative biomarkers detected.

The predicted biomarkers of both lymphoid and myeloid leukemias are reported in Additional file 2 (see section Additional files).

## Discussion

FunChisq measures the functional strength from row variable *X* to column variable *Y* in a contingency table via a model-free approach. Given the column sums, a contingency table maximizes the FunChisq statistic if and only if column variable *Y* is a non-constant mathematical function of row variable *X*. This theoretical optimality makes FunChisq model-free in promoting all forms of functional patterns regardless of parametric family, linearity, or monotonicity. This flexibility unconstrained by functional forms offers one a greater capacity in inferring causality with reduced biases than other methods.

The model-free property of FunChisq aligns well to the need of unbiased knowledge discovery in the analysis of vast uncharacterized human noncoding genes as uncovered by the FANTOM5 project, providing us a powerful instrument to assess objectively the evidence for a gene’s directional effect on human cancer.

## Conclusions

We have shown that the FunChisq statistical method is powerful in detecting directional association, sensitive to both monotonic and non-monotonic patterns. Strong functional patterns provide evidence for causality. Applying the method on the FANTOM5 data covering the largest number of potential noncoding genes for many cancer types, we revealed putative cancer driver ncRNAs with a directional effect on cancer status stronger than 90% of all 719 curated cancer genes. Furthermore, we predicted 332 potential cancer biomarkers for lymphoid leukemia and 79 for myeloid leukemia, stronger than all known lymphoid or myeloid leukemia genes. Our study thus contributes a catalog of novel biomarker candidates that may signify a deeper understanding of cancer biology.

## Methods

We used the normalized functional chi-squared test with an asymptotic normal null distribution to discover directional association in contingency tables [8, 11]. The test detects model-free functional dependency and does not need a prescribed functional form. The directional functional dependency can potentially indicate the causal direction of an interaction based on the causality-by-functionality principle [37].

An observed *r*×*c* contingency table *O* has *r* rows representing the discrete levels for independent variable and *c* columns representing the discrete levels for dependent variable. Let *O*_*ij*_ denote the sample counts at row *i* and column *j*. Let *O*_*i*·_ be the row sum of row *i* and *O*_·*j*_ be the column sum of column *j*, defined as

1 $$\begin{array}{@{}rcl@{}} O_{i\cdot} = \sum_{j=1}^{c}O_{ij} \quad \text{ and} \quad O_{\cdot j} = \sum_{i=1}^{r}O_{ij} \end{array} $$

Let *n* represent the sample size of table *O*. The FunChisq statistic of observed table *O* is defined by

2 $$\begin{array}{@{}rcl@{}} \chi_{f}^{2}(O) = \Bigg[\sum_{i=1}^{r}\sum_{j=1}^{c}\frac{(O_{ij} - O_{i\cdot} / c)^{2}}{O_{i\cdot} / c}\Bigg] - \sum_{j=1}^{c}\frac{(O_{\cdot j} - n/c)^{2}}{n/c} \end{array} $$

which asymptotically follows a chi-squared distribution with *ν*=(*r*−1)(*c*−1) degrees of freedom, under the null hypothesis of the row and column variables being statistically independent and an assumption of the dependent variable being uniformly distributed. We further define the normalized FunChisq by mean-shifting and standard-deviation-scaling $\chi _{f}^{2}(O)$ to

3 $$ \frac{\chi_{f}^{2}(O) - \nu}{\sqrt{2 \nu}} \quad \text{(Normalized FunChisq)}  $$

which asymptotically follows a standard normal distribution when the degrees of freedom *ν* is high [38] under the null hypothesis. Our empirical evaluation in Fig. 1 suggests that the normalized FunChisq is effective at detecting functional dependency even if *ν* is small.

We also introduce the function index *ξ*_*f*_ to measure the effect size of FunChisq test:

4 $$\begin{array}{@{}rcl@{}} \xi_{f} = \sqrt{\frac{\chi_{f}^{2}(O)}{n(c-1) - \sum\limits_{j=1}^{c}\frac{(O_{\cdot j} - n/c)^{2}}{n/c}}} \end{array} $$

The index assesses the strength of functional dependency of column variable *Y* on row variable *X*. It ranges from 0 to 1, with greater values representing stronger non-constant functionality. The index should be used in conjunction with the *p*-value of the test statistic to ensure both a sufficient effect and an acceptable statistical significance.

## Additional files

Additional file 1 FunChisq predicted 1108 putative cancer driver ncRNAs with stronger directional effect to cancer than 90% of 719 known cancer driver genes. (XLSX 70 kb)

Additional file 2 FunChisq predicted 332 potential cancer biomarkers for lymphoid leukemia and 79 for myeloid leukemia, which were stronger than 87 known lymphoid leukemia and 100 known myeloid leukemia driver genes. (XLSX 25 kb)

## Data Availability

See Additional files.

## Abbreviations

AML: Acute myeloid leukemia
CLL: Chronic lymphocytic leukemia
DREAM: Dialogue for reverse engineering assessments and methods
FANTOM5: Functional annotation of mammalian genome 5
FunChisq: Functional chi-squared test
HPN: Heritage provider network
ncRNA: Noncoding RNA
PR: Precision recall
ROC: Receiver operating characteristic
TCGA: The cancer genome atlas
TPM: Tags per million
TSS: Transcription start site

## About this supplement

This article has been published as part of *BMC Medical Genomics*, Volume 12 Supplement 7, 2019: Selected articles from the 14th International Symposium on Bioinformatics Research and Applications (ISBRA-18): medical genomics. The full contents of the supplement are available at https://bmcmedgenomics.biomedcentral.com/articles/supplements/volume-12-supplement-7v
